# *Burkholderia arboris* bacteremia initially identified as Burkholderia cepacia complex: a genome-based case report

**DOI:** 10.1007/s00203-026-05141-9

**Published:** 2026-08-13

**Authors:** Kazuhiro Itoh, Amane Tanaka, Daiki Maruno, Hiroshi Tsutani, Nozomi Otsuki, Atsushi Kuwata, Chiyo Kiriba, Yoshimasa Urasaki, Masamichi Ikawa, Hiromichi Iwasaki, Yasuhiko Mitsuke

**Affiliations:** 1 Department of Internal Medicine, NHO Awara National Hospital, 238-1 Kitagata, Awara, 910-4272 Fukui Japan; 2 Department of Community Health Science, Faculty of Medical Sciences, University of Fukui, Fukui, Japan; 3 Department of Laboratory Medicine, NHO Awara National Hospital, Awara, Japan

**Keywords:** Burkholderia cepacia complex, Bacteremia, Whole-genome sequencing, MALDI-TOF MS, Average nucleotide identity, Antimicrobial susceptibility testing

## Abstract

We report a bloodstream *Burkholderia arboris* isolate from a 75-year-old man without cystic fibrosis. The organism was recovered from both aerobic bottles of two separately collected blood-culture sets and was initially assigned to the Burkholderia cepacia complex (Bcc) by matrix-assisted laser desorption ionization–time-of-flight mass spectrometry. Whole-genome sequencing yielded three circular chromosomes and one circular plasmid. DFAST_QC identified *B. arboris* as the only type-strain match above the species threshold, with an average nucleotide identity of 99.48%; the next-highest match was *B. seminalis* at 93.33%. Multilocus sequence typing identified ST-2575, and ResFinder detected no acquired antimicrobial resistance genes. The patient improved after 14 days of meropenem therapy without recurrent *B. arboris* bacteremia. This report adds a clinically supported bloodstream infection, a complete genome resource, and detailed susceptibility data, while illustrating the importance of up-to-date reference genomes for species-level interpretation of unusual Bcc isolates.

**Supplementary Information:**

The online version contains supplementary material available at https://doi.org/10.1007/s00203-026-05141-9.

## Introduction

The Burkholderia cepacia complex (Bcc) comprises environmental non-fermenting Gram-negative bacilli that can cause healthcare-associated infections and bloodstream infections, particularly in vulnerable hosts (El Chakhtoura et al. 2017; Bressler et al. 2007; Brooks et al. 2019; Moehring et al. 2014). Routine matrix-assisted laser desorption ionization–time-of-flight mass spectrometry (MALDI-TOF MS) provides rapid and clinically useful identification, but species-level assignment within the complex may require sequence-based or whole-genome approaches, particularly for uncommon Bcc isolates (Depoorter et al. 2020).

*Burkholderia arboris* was formally described as a Bcc species in 2008 (Vanlaere et al. 2008). It has subsequently been identified among bloodstream isolates from patients without cystic fibrosis and among patient and contaminated-product isolates in a healthcare-associated outbreak (Chien et al. 2018; Bender et al. 2022). However, clinically and genomically detailed reports remain limited. Here, we describe a bloodstream isolate from an older non-cystic fibrosis patient in Japan that was initially identified as Bcc by routine MALDI-TOF MS and subsequently resolved as *B. arboris* by genome-based analysis. This report provides a complete circularized genome, MLST sequence type ST-2575, detailed antimicrobial susceptibility results, and an example of how the availability of comprehensive and up-to-date type-strain genomes can affect species-level interpretation.

## Materials and methods

### Study design and rationale for genome sequencing

This was a single-isolate microbiological characterization study of a bloodstream *Burkholderia* isolate recovered from a patient without cystic fibrosis. The isolate was included because it was recovered from blood cultures, was initially assigned only to the Burkholderia cepacia complex by routine MALDI-TOF MS identification, and was obtained from a clinical context in which species-level characterization was considered relevant. No additional patient isolates were included. WGS was performed after completion of clinical management for species-level characterization and taxonomic assessment, not as part of routine diagnostic workflow or immediate treatment decision-making.

### Routine microbiological identification and antimicrobial susceptibility testing

Routine microbiological identification and antimicrobial susceptibility testing were performed using the bloodstream isolate recovered from positive blood culture bottles. The isolate was assigned to the Bcc by MALDI-TOF MS using the BD Bruker MALDI Biotyper sirius system (Becton Dickinson Japan, Tokyo, Japan) and Bruker MALDI Biotyper Library BDAL version 13. The two aerobic bottle-derived isolates yielded top database matches to *B. cepacia* and *B. pyrrocinia*, respectively, both with identification scores of 2.16; *B. pyrrocinia* is included in the Bcc. Antimicrobial susceptibility testing was performed using Dry Plate DP192 (Eiken Chemical Co., Ltd., Tokyo, Japan). Quality control was performed using *Escherichia coli* ATCC 25,922 and *Pseudomonas aeruginosa* ATCC 27,853, and QC results were within CLSI-recommended ranges. The testing laboratory referred to CLSI M100, 35th edition (Clinical and Laboratory Standards Institute 2025), for susceptibility interpretation. Because Bcc-specific MIC breakpoints were no longer included in that edition, historical Bcc-specific MIC breakpoints from the 33rd edition (Clinical and Laboratory Standards Institute 2023) were used for ceftazidime, meropenem, levofloxacin, minocycline, and trimethoprim-sulfamethoxazole. For agents without Bcc-specific MIC breakpoints, criteria for other non-Enterobacterales in the 35th edition were used only as supplementary interpretive guidance. The corresponding laboratory-reported categories were retained only as supplementary information, and raw MIC values were prioritized.

### Whole-genome sequencing and bioinformatics analysis

Genomic DNA was extracted using the magLEAD MagDEA Dx SV PS protocol. Libraries were prepared using the Rapid Barcoding Kit 24 V14 and sequenced on a MinION sequencer (Oxford Nanopore Technologies) with an R10.4.1 flow cell. Reads were basecalled using Dorado v1.1.1 and trimmed and quality-filtered using fastplong v0.4.1 with a minimum quality score of Q15 and a minimum read length of 5,000 bp. De novo assembly was performed using Flye v2.9.6 (Kolmogorov et al. 2019) in --nano-corr mode. No estimated genome size or --asm-coverage value was specified, and all other parameters were left at their default settings. The assembly was polished using medaka with the dna_r10.4.1_e8.2_400bps_sup@v5.0.0 model.

Genome annotation was performed using the DFAST web service (Tanizawa et al. 2018) and the NCBI Prokaryotic Genome Annotation Pipeline (Tatusova et al. 2016) after sequence deposition. Genome completeness and contamination were assessed using CheckM (Parks et al. 2015). ANI-based taxonomic reassessment was performed on 24 July 2026 using DFAST_QC v1.1.1 (Elmanzalawi et al. 2025) with the full reference dataset version 2026-04-20. The DFAST_QC workflow first screened reference genomes using Mash, selected the 10 closest candidate genomes, and then calculated ANI and aligned fractions using skani (Shaw and Yu 2023). Species-level interpretation was based on the species-specific ANI threshold implemented in DFAST_QC.

Representative 16 S rRNA gene sequences were compared with reference sequences using NCBI BLASTn. In silico MLST was performed using the PubMLST Bcc seven-locus scheme (Baldwin et al. 2005), accessed on 4 November 2025 and rechecked in July 2026. Acquired antimicrobial resistance genes were screened using ResFinder-2.5.1 (Zankari et al. 2012), accessed on 4 November 2025, with thresholds of 90.0% nucleotide identity and 60.0% minimum gene-length coverage.

The circular plasmid sequence was compared with publicly available nucleotide sequences using NCBI BLASTn, and query coverage and nucleotide identity were reviewed for the highest-scoring plasmid and chromosomal matches. The whole-genome shotgun sequencing project has been deposited at DDBJ/ENA/GenBank under accession number JBXXZJ000000000.1. The associated BioProject and BioSample accession numbers are PRJNA1461421 and SAMN58218997, respectively.

### Clinical source and microbiological characterization

A 75-year-old man residing in a nursing facility was transferred to the emergency department of NHO Awara National Hospital, Awara, Fukui, Japan, with persistent fever and nausea. He was diagnosed with facioscapulohumeral muscular dystrophy 30 years earlier and had become nearly bedridden and fully dependent for activities of daily living. His medical history included type 2 diabetes mellitus and hypertension, and insulin therapy had been discontinued approximately one month before presentation because of poor oral intake and progressive disuse syndrome. He developed fever the day before admission and received a single 1 g dose of cefepime from his primary physician; however, the fever persisted, and nausea developed.

On admission, his temperature was 38.0 °C; blood pressure, 170/91 mmHg; heart rate, 97 beats/min; respiratory rate, 25 breaths/min; oxygen saturation, 96% on room air; and Glasgow Coma Scale score, 15. The patient was hemodynamically stable and did not require vasopressors. Bilateral pitting edema was observed. Laboratory tests showed a leukocyte count of 9,700/µL with 90% neutrophils, a hemoglobin level of 9.1 g/dL, a platelet count of 90 × 10³/µL, albumin 2.2 g/dL, creatinine 1.05 mg/dL, glucose 220 mg/dL, C-reactive protein 12.74 mg/dL, and procalcitonin > 10.0 ng/mL. Coagulation tests showed mildly elevated fibrin/fibrinogen degradation products and reduced antithrombin activity. Computed tomography and magnetic resonance imaging did not reveal pneumonia, intra-abdominal abscess, spondylodiscitis, or another focal infectious source, and transthoracic echocardiography showed no valvular vegetation. No central venous catheter, urinary catheter, tracheostomy, or gastrostomy tube was present.

**Fig. 1:**
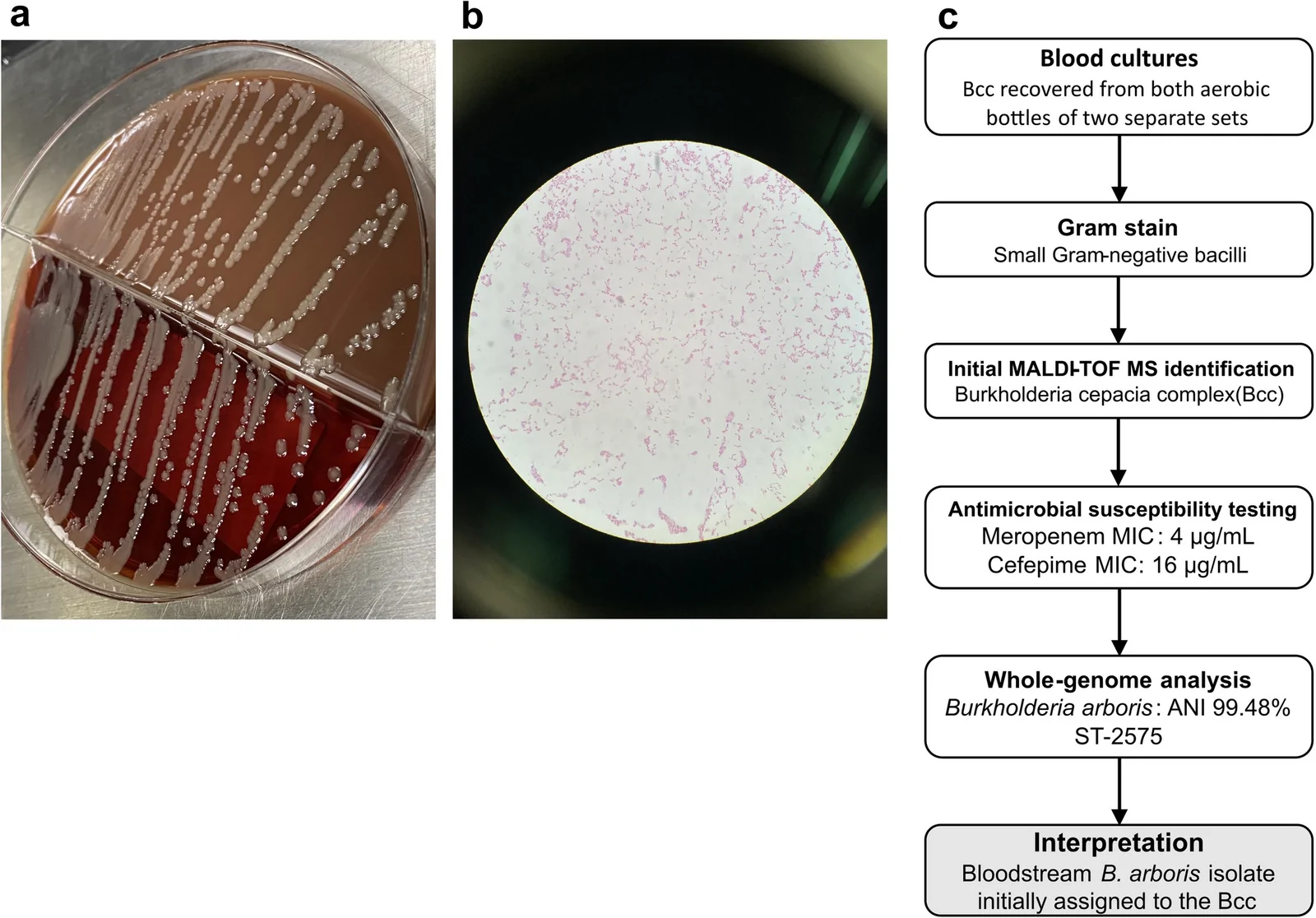
Microbiological characteristics and diagnostic workflow of the bloodstream isolate. **a** Colony morphology on a biplate containing CA-supplemented 5% sheep blood agar with colistin and aztreonam on the red side and vancomycin-supplemented chocolate agar on the brown side. **b** Gram stain showing small Gram-negative bacilli (original magnification, ×1000). **c** Diagnostic workflow. The isolate was recovered as Bcc from both aerobic bottles of two separately collected blood-culture sets and was initially assigned to the Bcc by MALDI-TOF MS. DFAST_QC-based ANI analysis identified the isolate as *B. arboris* with an ANI of 99.48%, while MLST identified ST-2575

Two sets of blood cultures, each consisting of one aerobic and one anaerobic bottle, were obtained before meropenem administration. Meropenem (0.5 g) was administered every 8 h, and the patient became afebrile on day 2. The Bcc organism was recovered from both aerobic bottles of the two separately collected blood-culture sets. One anaerobic bottle yielded coagulase-negative staphylococci, whereas the other anaerobic bottle remained negative. The exact time to positivity in hours was unavailable. Quantitative bacterial load was not assessed. Gram staining of the positive aerobic bottles showed small Gram-negative bacilli. MALDI-TOF MS assigned the two aerobic bottle-derived isolates to the Bcc, with top database matches to *B. cepacia* and *B. pyrrocinia*, both with scores of 2.16. Upon subculture, all visible colonies growing on the biplate were assigned to the Bcc (Fig. 1a).

The principal microbiological and genome-based characteristics of the isolate are summarized in Table 1. Phenotypic antimicrobial susceptibility testing showed MICs of 4 µg/mL for meropenem and 16 µg/mL for cefepime. Full MIC results, laboratory-reported categories, and the breakpoint basis for interpretation are provided in Supplementary Table S1. Meropenem was administered for 14 days with sustained clinical improvement.

Edema had been noted before admission. Approximately 6 months before admission, the serum albumin level was 3.5 g/dL and the creatinine level was 0.64 mg/dL; however, no pre-admission quantitative urinary protein data were available, and no renal disorder had previously been diagnosed. On admission, the albumin level was 2.2 g/dL, the creatinine level was 1.05 mg/dL, and urinary dipstick testing showed proteinuria of ≥ 300 mg/dL. During hospitalization, the albumin level decreased to 1.8 g/dL, and 24-h urinary protein excretion was 2.57 g/day. A subsequent urine protein-to-creatinine ratio was 13.88. These findings demonstrated marked proteinuria and hypoalbuminemia, but they did not establish whether the renal abnormality was pre-existing, exacerbated by the acute illness, or both.

On day 28, he developed a separate episode of extended-spectrum β-lactamase-producing *Escherichia coli* urinary tract infection. No recurrence of *Burkholderia* bacteremia occurred before discharge.

### Genome-based characterization

The final assembly represented a complete circularized genome comprising three circular chromosomal replicons and one circular plasmid. The chromosomal replicons were 3,577,940 bp, 3,419,015 bp, and 1,253,950 bp in length, and the plasmid pMTY26381 was 97,736 bp in length, giving a total genome size of 8,348,641 bp. The assembly consisted of four contigs/scaffolds, with an N50 of 3,419,015 bp. The genomic GC content was 67.0%. The sequencing run generated 32,359 reads, with a read N50 of 13,128 bp and 171,485,598 total bases. The nominal raw-read depth, calculated by dividing the total number of deposited bases by the assembly size, was approximately 20.5×. NCBI PGAP annotation identified 7,582 genes, including 7,491 CDSs, 91 RNA genes, six complete copies each of the 5 S, 16 S, and 23 S rRNA genes, 69 tRNAs, and four ncRNAs. CheckM analysis showed 100% completeness and 0.48% contamination.

Representative 16 S rRNA gene copies showed the highest BLASTn similarity to *Burkholderia lata* strain 383 (NR_102890.1), with 99.67% identity and 100% query coverage. Multiple recognized Bcc species showed similarly high 16 S rRNA gene sequence similarity, confirming that 16 S rRNA analysis alone was insufficient for species-level discrimination within the Bcc (Kim et al. 2014).

DFAST_QC v1.1.1, using the full reference dataset version 2026-04-20, screened reference genomes using Mash and selected the 10 closest candidate genomes for ANI analysis with skani. *Burkholderia arboris* GCA_902499125.1 was the only candidate with an ANI above the applicable 95% species threshold. The ANI was 99.48%, with aligned fractions of 95.86% for the reference genome and 94.97% for the query genome. The DFAST_QC taxonomic result was classified as conclusive.

The next-highest type-strain match was *B. seminalis* LMG 24,067 (GCA_902832885.1), with an ANI of 93.33% and aligned fractions of 68.34% for the reference and 65.25% for the query. Thus, the ANI difference between the best and second-best type-strain matches was 6.15% points, providing a clear margin for species-level assignment as *B. arboris*.

In silico MLST using the PubMLST Bcc seven-locus scheme identified the allelic profile *atpD* 169, *gltB* 137, *gyrB* 304, *recA* 149, *lepA* 316, *phaC* 154, and *trpB* 146. This profile was assigned ST-2575 in the PubMLST Bcc database. ResFinder-2.5.1 detected no acquired antimicrobial resistance genes at the applied thresholds of 90.0% nucleotide identity and 60.0% minimum gene-length coverage.

The 97,736-bp circular plasmid pMTY26381 contained 98 predicted CDSs. Annotation identified putative replication initiation and partition-associated proteins, including ParA and ParB, together with multiple integrase- and transposase-related proteins and a predicted HicB-family antitoxin. No acquired antimicrobial resistance gene or obvious complete conjugation machinery was identified.

BLASTn analysis did not identify a close full-length plasmid counterpart. The highest-coverage plasmid match was *Burkholderia multivorans* plasmid p1_2016Y70888756VI (CP090706.1), which covered 35% of pMTY26381 at 98.50% nucleotide identity. A region of approximately 31.2 kb also showed approximately 99.47% identity to chromosome 3 of *B. arboris* LMG 24,066, suggesting a mosaic structure containing sequences related to both plasmid and chromosomal regions of Bcc organisms.

## Discussion

This report describes a clinically significant bloodstream infection caused by *B. arboris* in a patient without cystic fibrosis. The organism was recovered from both aerobic bottles of two separately collected blood-culture sets obtained before meropenem administration. The patient had fever, markedly elevated inflammatory markers, and prompt clinical improvement after meropenem therapy, supporting the interpretation of true bacteremia rather than contamination. However, these findings establish clinical significance in this patient and do not independently demonstrate species-specific virulence of *B. arboris*. The reason why *B. arboris* was recovered only from the aerobic bottles could not be determined because the exact time to positivity and quantitative bacterial load were unavailable. Although the single cefepime dose administered before blood-culture collection may have affected overall culture recovery, there is no evidence that it specifically accounted for the difference between the aerobic and anaerobic bottles.

Bcc bloodstream infection is uncommon in patients without cystic fibrosis but has been reported in chronically ill, hospitalized, and device-exposed patients (El Chakhtoura et al. 2017; Bressler et al. 2007). In the present patient, severe functional impairment due to facioscapulohumeral muscular dystrophy, diabetes mellitus, hypoalbuminemia, and proteinuria were considered markers of host vulnerability rather than proven Bcc-specific risk factors. Edema had been present before admission, and proteinuria and hypoalbuminemia were already evident on admission. However, pre-admission quantitative proteinuria data were unavailable, and no previous diagnosis of nephrotic syndrome or chronic kidney disease had been documented. Therefore, whether the renal abnormalities represented a pre-existing process, acute illness-related exacerbation, or both could not be determined. They should not be interpreted as an established causal risk factor for bacteremia.

Clinical evidence specifically concerning *B. arboris* remains limited. In a Taiwanese cohort of 54 non-cystic fibrosis patients with Bcc bacteremia, one blood isolate was identified as *B. arboris* by *recA* sequencing, demonstrating that *B. arboris* bacteremia has previously been documented (Chien et al. 2018). In addition, genome-based investigation of a nationwide healthcare-associated outbreak in Germany identified a *B. arboris* cluster comprising patient, mouthwash, and manufacturer-derived isolates; the associated clinical events included both colonization and putative infection (Bender et al. 2022). The present case therefore does not represent the first reported clinical isolation of *B. arboris*, but adds a clinically supported bloodstream infection with a complete genome, detailed susceptibility results, treatment course, and outcome.

From a diagnostic standpoint, this case illustrates both the utility and limitations of routine MALDI-TOF MS for uncommon Bcc isolates. MALDI-TOF MS provided a clinically actionable Bcc-level identification, but species-level resolution required genome-based analysis. Because Bcc-specific MIC breakpoints were available in CLSI M100 Ed33 but were no longer included in Ed35 (Clinical and Laboratory Standards Institute 2023, 2025), the raw MIC values and breakpoint notes in Supplementary Table S1 should be prioritized over categorical interpretations for agents without Bcc-specific criteria. The patient received only a single 1-g dose of cefepime before admission; therefore, its clinical efficacy could not be evaluated, and persistent fever after that dose should not be interpreted as treatment failure. The cefepime MIC was 16 µg/mL, whereas the meropenem MIC was 4 µg/mL. The subsequent response to meropenem illustrates the practical importance of early susceptibility-guided antimicrobial selection when Bcc bloodstream infection is suspected, without proving that cefepime would have been clinically ineffective.

ResFinder detected no acquired antimicrobial resistance genes under the applied thresholds. However, this finding does not exclude intrinsic or mutation-associated resistance. Bcc organisms may exhibit reduced susceptibility through a low-permeability cell envelope, multidrug efflux systems, chromosomally encoded β-lactamases, alterations in penicillin-binding proteins or other drug targets, and outer-membrane characteristics that reduce aminoglycoside activity (Rhodes and Schweizer 2016; Podnecky et al. 2015; Everaert and Coenye 2016). These mechanisms may help explain the elevated MICs for several β-lactams and aminoglycosides despite the absence of acquired resistance genes. Because β-lactamase expression, efflux activity, membrane permeability, and target mutations were not functionally investigated in the present isolate, the contribution of individual mechanisms could not be determined.

The key genomic finding was the species-level resolution of the isolate as *B. arboris*. DFAST_QC v1.1.1, using the full reference dataset version 2026-04-20, selected 10 candidate type-strain genomes by Mash and calculated ANI and aligned fractions using skani. *Burkholderia arboris* GCA_902499125.1 was the only candidate above the applicable 95% species threshold, with an ANI of 99.48%; the next-highest match, *B. seminalis* LMG 24,067, showed an ANI of 93.33%. This clear margin supported the species-level assignment.

The reference and query aligned fractions of 95.86% and 94.97%, respectively, represent the proportions of genomic fragments contributing to orthologous mappings and should not be interpreted as estimates of assembly completeness. The unmatched fractions may include plasmid sequences and other accessory or highly divergent regions, but the specific regions were not resolved by the summary taxonomy-check output. The DFAST_QC reference assembly GCA_902499125.1 is a contig-level assembly comprising 83 contigs rather than a complete closed genome. However, an independent comparison with the complete *B. arboris* type-strain assembly GCA_984667665.1 yielded the same ANI of 99.48%, with aligned fractions of 95.10% for the type-strain assembly and 95.78% for the query, supporting the robustness of the species-level assignment.

Initial analysis using an earlier reference set had not identified a recognized species above the conventional ANI threshold. The revised interpretation therefore illustrates that genome-based taxonomy depends not only on sequencing quality and analytical methods, but also on the availability of comprehensive and up-to-date reference genomes and regular database updates.

The nominal raw-read depth of approximately 20.5× was sufficient to generate a complete circularized assembly for ANI and MLST analyses. However, this depth was relatively modest for confident detection of low-frequency small variants, which was not an objective of the present study.

The 16 S rRNA gene analysis supported placement within the Bcc but was insufficient for species-level discrimination because multiple recognized Bcc species showed similarly high sequence similarity. MLST identified the allelic profile *atpD* 169, *gltB* 137, *gyrB* 304, *recA* 149, *lepA* 316, *phaC* 154, and *trpB* 146. This profile was assigned ST-2575 in the PubMLST Bcc database. 

The 97,736-bp circular plasmid pMTY26381 encoded predicted replication- and partition-associated proteins together with multiple integrase- and transposase-related proteins. BLASTn did not identify a close full-length plasmid counterpart; the highest-coverage plasmid hit covered only 35% of pMTY26381 at 98.50% identity. Different regions also showed high similarity to chromosomal and plasmid sequences from other Bcc members. No acquired antimicrobial resistance genes or obvious complete conjugation machinery were identified, and the mobility and biological significance of this replicon remain uncertain.

The source of bacteremia remained undetermined. Bcc organisms have caused outbreaks and pseudo-outbreaks through contaminated healthcare products and can persist in aqueous environmental reservoirs (Tavares et al. 2020; Brooks et al. 2019; Moehring et al. 2014; Bender et al. 2022). No intravascular or urinary device, tracheostomy, gastrostomy tube, known contaminated-product exposure, or institutional cluster was identified in this case. Environmental sampling was not performed; therefore, community, nursing-facility, healthcare-associated, and environmental acquisition could not be distinguished.

This case provides three practical lessons. First, recovery of a Bcc organism from separately collected blood-culture sets should not automatically be dismissed as contamination when the clinical and inflammatory findings support bacteremia. Second, Bcc-level MALDI-TOF MS identification may be sufficient for initial management, but WGS should be considered when species-level resolution is epidemiologically relevant, the clinical context is unusual, or routine identification is discordant or incomplete. Third, antimicrobial selection should be guided primarily by measured MICs and clearly documented breakpoint limitations. The present report adds a clinically supported *B. arboris* bloodstream infection, a complete genome resource, a newly assigned MLST sequence type (ST-2575), and detailed susceptibility data without claiming species-specific virulence or first occurrence.

**Table 1:** Microbiological and genome-based characterization of the bloodstream isolate

Parameter	Result
Routine microbiology	
Initial identification	Assigned to the Bcc by MALDI-TOF MS using the BD Bruker MALDI Biotyper sirius system and BDAL version 13; top matches were *B. cepacia* and *B. pyrrocinia*, both with scores of 2.16
Blood cultures	Bcc was recovered from both aerobic bottles of two separately collected blood-culture sets; one anaerobic bottle yielded coagulase-negative staphylococci and the other remained negative
Gram stain	Small Gram-negative bacilli
Antimicrobial susceptibility testing	
Method and quality control	Dry Plate DP192; *E. coli* ATCC 25,922 and *P. aeruginosa* ATCC 27,853 yielded results within CLSI-recommended QC ranges
Key MICs	Meropenem, 4 µg/mL; cefepime, 16 µg/mL. Full MIC results and breakpoint handling are provided in Supplementary Table S1
Genome-based characterization	
Assembly	Complete circularized genome comprising three chromosomes and one plasmid; total size, 8,348,641 bp; N50, 3,419,015 bp; GC content, 67.0%; nominal raw-read depth, approximately 20.5×
Annotation and quality	7,582 genes, including 7,491 CDSs and 91 RNA genes; CheckM completeness, 100%; contamination, 0.48%
ANI-based assignment	DFAST_QC v1.1.1 identified *B. arboris* GCA_902499125.1 as the closest type-strain genome: ANI, 99.48%; reference aligned fraction, 95.86%; query aligned fraction, 94.97%
Next-highest ANI match	*B. seminalis* LMG 24,067, GCA_902832885.1: ANI, 93.33%
16 S rRNA comparison	Highest BLASTn similarity to *B. lata* strain 383: 99.67% identity and 100% query coverage; insufficient for species-level discrimination within the Bcc
MLST	ST-2575: *atpD* 169, *gltB* 137, *gyrB* 304, *recA* 149, *lepA* 316, *phaC* 154, and *trpB* 146
Acquired resistance genes	None detected by ResFinder-2.5.1 under the applied identity and gene-length coverage thresholds
Circular plasmid	pMTY26381, 97,736 bp; predicted replication/partition and mobile-element-associated proteins; no acquired antimicrobial resistance genes or close full-length plasmid counterpart identified
Interpretation	
Final interpretation	Bloodstream *B. arboris* isolate initially assigned to the Bcc by routine MALDI-TOF MS

ANI-based taxonomic reassessment was performed using DFAST_QC v1.1.1 with the full reference dataset version 2026-04-20; ANI and aligned fractions were calculated using skani. In silico MLST was performed using the PubMLST Bcc seven-locus scheme. Acquired antimicrobial resistance genes were screened using ResFinder-2.5.1 with thresholds of 90.0% nucleotide identity and 60.0% minimum gene-length coverage. Full antimicrobial susceptibility results and breakpoint handling are provided in Supplementary Table S1. ANI, average nucleotide identity; Bcc, Burkholderia cepacia complex; CLSI, Clinical and Laboratory Standards Institute; MALDI-TOF MS, matrix-assisted laser desorption ionization time-of-flight mass spectrometry; MIC, minimum inhibitory concentration; MLST, multilocus sequence typing; QC, quality control

## Supplementary Information

Below is the link to the electronic supplementary material.

Supplementary Material 1

## Data Availability

The clinical data underlying this report are not publicly available because they contain information that may compromise patient privacy. The whole-genome shotgun sequencing project is available at DDBJ/ENA/GenBank under master accession JBXXZJ000000000.1. The corresponding genome assembly is available under accession GCA_057369595.1, with BioProject PRJNA1461421, BioSample SAMN58218997, and SRA run SRR38416443. The three chromosome component sequences are available under accessions CM170668.1, CM170669.1, and CM170670.1. The 97,736-bp circular plasmid component pMTY26381 is available within the WGS project under component accession JBXXZJ010000004.1.

## Abbreviations

ANI: Average nucleotide identity
Bcc: Burkholderia cepacia complex
CLSI: Clinical and laboratory standards institute
MALDI-TOF MS: Matrix-assisted laser desorption ionization–time-of-flight mass spectrometry
MIC: Minimum inhibitory concentration
MLST: Multilocus sequence typing
PGAP: Prokaryotic genome annotation pipeline
QC: Quality control
WGS: Whole-genome sequencing
