# Supplementary material for: Burkholderia arboris bacteremia initially identified as Burkholderia cepacia complex: a genome-based case report
Source: Arch Microbiol. 2026 Aug 13;208(11):577. doi: 10.1007/s00203-026-05141-9 (PMC13473173; doi:10.1007/s00203-026-05141-9)
Supplement: Supplementary file 1 [file 203_2026_5141_MOESM1_ESM.docx]

**Supplementary Table S1. Antimicrobial susceptibility testing included 20 agents. Historical Bcc-specific MIC breakpoints from CLSI M100 Ed33 were applied to five agents: ceftazidime, meropenem, levofloxacin, minocycline, and trimethoprim-sulfamethoxazole. Bcc-specific MIC breakpoints were removed from CLSI M100 beginning with Ed34. For the remaining 15 agents, laboratory-reported categories were based on the Other Non-Enterobacterales guidance in CLSI M100 Ed35, used only as supplementary interpretive guidance. These categories should be interpreted cautiously, and raw MIC values should be prioritized.**

| **Antimicrobial agent** | **MIC (μg/mL)** | **Laboratory-reported category** | **Breakpoint basis / handling** | **Comment** |
| --- | --- | --- | --- | --- |
| Ceftazidime | ≤4 | S | Historical Bcc-specific MIC breakpoint, CLSI M100 Ed33 | Bcc-specific MIC breakpoint from CLSI M100 Ed33. |
| Meropenem | 4 | S | Historical Bcc-specific MIC breakpoint, CLSI M100 Ed33 | Clinically used definitive therapy. |
| Levofloxacin | 0.5 | S | Historical Bcc-specific MIC breakpoint, CLSI M100 Ed33 |  |
| Minocycline | ≤4 | S | Historical Bcc-specific MIC breakpoint, CLSI M100 Ed33 |  |
| Trimethoprim-sulfamethoxazole | ≤40 | S | Historical Bcc-specific MIC breakpoint, CLSI M100 Ed33 | MIC shown as reported by the testing system. |
| Piperacillin | 32 | R | CLSI M100 Ed35 Other Non-Enterobacterales guidance; supplementary interpretation only | No current Bcc-specific MIC breakpoint; interpret cautiously. |
| Piperacillin-tazobactam | 32 | I | CLSI M100 Ed35 Other Non-Enterobacterales guidance; supplementary interpretation only | No current Bcc-specific MIC breakpoint; interpret cautiously. |
| Cefepime | 16 | I | CLSI M100 Ed35 Other Non-Enterobacterales guidance; supplementary interpretation only | No current Bcc-specific MIC breakpoint; interpret cautiously. |
| Ceftriaxone | >32 | R | CLSI M100 Ed35 Other Non-Enterobacterales guidance; supplementary interpretation only | No current Bcc-specific MIC breakpoint; interpret cautiously. |
| Imipenem-cilastatin | 8 | I | CLSI M100 Ed35 Other Non-Enterobacterales guidance; supplementary interpretation only | No current Bcc-specific MIC breakpoint; interpret cautiously. |
| Gentamicin | >8 | R | CLSI M100 Ed35 Other Non-Enterobacterales guidance; supplementary interpretation only | No current Bcc-specific MIC breakpoint; interpret cautiously. |
| Amikacin | 32 | I | CLSI M100 Ed35 Other Non-Enterobacterales guidance; supplementary interpretation only | No current Bcc-specific MIC breakpoint; interpret cautiously. |
| Ciprofloxacin | 1 | S | CLSI M100 Ed35 Other Non-Enterobacterales guidance; supplementary interpretation only | No current Bcc-specific MIC breakpoint; interpret cautiously. |
| Ampicillin | >16 | R | CLSI M100 Ed35 Other Non-Enterobacterales guidance; supplementary interpretation only | No current Bcc-specific MIC breakpoint; interpret cautiously. |
| Ampicillin-sulbactam | >16 | R | CLSI M100 Ed35 Other Non-Enterobacterales guidance; supplementary interpretation only | No current Bcc-specific MIC breakpoint; interpret cautiously. |
| Cefazolin | >4 | R | CLSI M100 Ed35 Other Non-Enterobacterales guidance; supplementary interpretation only | No current Bcc-specific MIC breakpoint; interpret cautiously. |
| Cefotiam | >16 | R | CLSI M100 Ed35 Other Non-Enterobacterales guidance; supplementary interpretation only | No current Bcc-specific MIC breakpoint; interpret cautiously. |
| Cefmetazole | >32 | R | CLSI M100 Ed35 Other Non-Enterobacterales guidance; supplementary interpretation only | No current Bcc-specific MIC breakpoint; interpret cautiously. |
| Cefcapene pivoxil | >2 | R | CLSI M100 Ed35 Other Non-Enterobacterales guidance; supplementary interpretation only | No current Bcc-specific MIC breakpoint; interpret cautiously. |
| Cefoperazone-sulbactam | >32 | R | CLSI M100 Ed35 Other Non-Enterobacterales guidance; supplementary interpretation only | No current Bcc-specific MIC breakpoint; interpret cautiously. |

Notes: MIC values are shown in μg/mL. AST was performed using Dry Plate DP192. Quality control was performed using *Escherichia coli* ATCC 25922 and *Pseudomonas aeruginosa* ATCC 27853, and the results were within CLSI-recommended ranges. Historical Bcc-specific MIC breakpoints from CLSI M100 Ed33 were applied to ceftazidime, meropenem, levofloxacin, minocycline, and trimethoprim-sulfamethoxazole. Bcc-specific MIC breakpoints were removed beginning with CLSI M100 Ed34. For the remaining agents, the listed S/I/R categories reproduce the categories reported by the testing laboratory using the Other Non-Enterobacterales guidance in CLSI M100 Ed35 as supplementary interpretive guidance. These categories should not be interpreted as Bcc-specific CLSI categorizations. Raw MIC values should be given priority.

Abbreviations: AST, antimicrobial susceptibility testing; Bcc, Burkholderia cepacia complex; CLSI, Clinical and Laboratory Standards Institute; I, intermediate; MIC, minimum inhibitory concentration; QC, quality control; R, resistant; S, susceptible.
